# Recognizing Trauma-related Dissociation Behind Psychotic Phenomena

**DOI:** 10.31083/AP46069

**Published:** 2026-06-02

**Authors:** Hong Wang Fung, Wai Tong Chien

**Affiliations:** ^1^School of Nursing, The Hong Kong Polytechnic University, Hung Hom, Kowloon, Hong Kong SAR, China; ^2^Mental Health Research Centre, The Hong Kong Polytechnic University, Hung Hom, Hong Kong SAR, China; ^3^Nethersole School of Nursing, Faculty of Medicine, The Chinese University of Hong Kong, Shatin, N.T., Hong Kong SAR, China

Psychosis is a significant public health concern and a clinical challenge. However, some psychotic(-like) symptoms or phenomena may be better explained by trauma-related dissociative processes. In such cases, the clinical implications are significant, as trauma-related dissociation requires treatments that are different from those for genuine psychosis. Recent clinical research evidence has suggested that there are at least five important facts about the relationship between psychosis and trauma-related dissociative processes. They include: (a) individuals with dissociative identity disorder report statistically significantly more positive symptoms (of psychosis) than do those with schizophrenia [1, 2]; (b) positive symptoms such as auditory verbal hallucinations (AVHs) are common in people with complex dissociative disorders [3]; (c) the relationship between psychosis and dissociation is obvious – as revealed in a meta-analysis, there is a robust relationship between dissociation and positive psychotic symptoms across clinical and nonclinical studies [4]; (d) psychotic symptoms may be predicted by dissociation and its symptoms [5]; and, (e) unrecognized trauma and dissociation are common in people with a psychotic disorder [6, 7]. Some people who have psychotic disorders may actually have dissociation; and it is sometimes referred to as dissociative psychosis, although this condition has not been officially recognized in diagnostic manuals [8].

In this paper, we suggest a few reasons why trauma-related dissociative processes or symptoms may be misidentified as genuine psychotic symptoms. These highlight the importance of assessing trauma and dissociation in individuals presenting with psychotic(-like) symptoms. Table 1 summarizes the eight possible links between psychotic-like symptoms and dissociative disorders.

**Table 1.** **Dissociative processes or phenomena that may be misidentified as genuine psychotic symptoms**.

1. Flashbacks of trauma-related memories, sensations, or emotions
2. Internal communication among different dissociated self-states
3. Intrusions from other dissociated self-states
4. Depersonalization and derealization
5. Vivid inner world
6. Visions or voices “produced” by dissociated self-states
7. The delusions of being a separate person
8. Some alter personality states that are psychotic

## 1. Flashbacks of Trauma-related Memories, Sensations, or Emotions 

Flashbacks involving trauma-related memories, sensations, or emotions are common among traumatized and dissociative individuals. Flashbacks, a hallmark symptom of post-traumatic stress disorder (PTSD), are also dissociative in nature. During a flashback, individuals may hear voices, see visions, or experience sensations related to the traumatic events, although the link between the contents and the traumatic events may be less than clear. For example, they may hear an abuser’s words or witness a horrific scene, as if it were happening again. If clinicians fail to recognize the connection between these symptoms and the individuals’ trauma history, flashbacks may be mislabeled as psychotic symptoms. Although flashbacks themselves are not psychotic, in extreme cases, individuals may lose intact reality testing, presenting with what appears to be a genuine psychotic episode. However, these episodes are better understood as trauma-related dissociative processes. In such cases, it is important to help the person feel safe, remove triggers, implement safety measures, and ground them in the present moment, reassuring them that they are safe now and that the event only happened in the past.

## 2. Internal Communication Among Different Dissociated Self-states

People with dissociative disorders, particularly those with dissociative identity disorder or its partial form, typically have distinct, dissociated self-states or identities, sometimes referred to as ‘alters’. Most individuals with dissociative identity disorder are aware of these dissociated self-states [9, 10]. Therefore, they may hear how ‘other alters’ (alternative identities within a person) say or see their actions, reflecting internal communication among these self-states. Such experiences should not be mistaken for psychotic symptoms. At times, this communication can be distressing, as some alters may criticize the person or command actions such as self-harm, which may resemble Schneiderian first-rank symptoms (e.g., voices arguing or commenting). However, these are dissociative phenomena. In such cases, it is essential to foster healthy and peaceful internal communication, resolve internal conflicts, and address the needs of specific self-states. One can imagine that the process is like providing group counseling and mediation within a single individual.

## 3. Intrusions From Other Dissociated Self-states (e.g., Made Thoughts, Made Impulses, Made Volition, Made Feelings, “Seeing” an Alter)

Dissociation is not often successful, and therefore partial dissociation may actually be more common than full dissociation [9]. Dissociative intrusions are common among traumatized and dissociative individuals. They may experience intrusive thoughts, emotions, or impulses, and may sometimes act or speak in ways they do not intend. They may feel influenced by an unknown force, which could be a fully developed dissociated self-state (e.g., with a distinct name and personality) or a less defined self-state that is not as distinct as a separate alter. In some cases, they may actually “see” an alter (e.g., a big man or a little girl) without realizing it is in fact their internal self-state, which can resemble psychotic hallucinations. These intrusions may be mistaken for psychotic symptoms but are, in fact, dissociative in nature.

## 4. Depersonalization and/or Derealization Experiences

Depersonalization and derealization are two major symptoms of dissociation [11]. To meet the diagnostic criteria for depersonalization-derealization disorder, individuals must maintain intact reality testing. During depersonalization or derealization episodes, they recognize that their experiences are not objectively “real”, indicating reasonable insights and intact reality testing. However, when describing these experiences, some may report feeling detached from parts of their body, feeling like a robot, perceiving a family member as strange or unfamiliar, or viewing their surroundings (e.g., home or workplace) as odd, blurred, or colorless. These symptoms should not be mistaken for psychotic phenomena.

## 5. Vivid Internal World

People with dissociative identity disorder often have a vivid internal world in which different “things” or “people” can exist, such as a garden, a castle, or unfamiliar people. This internal world can interact with the external world. For example, extreme stress in the real world may metaphorically disrupt the internal world, manifesting as storms or damaged houses. These internal-world experiences should not be mistaken for psychotic symptoms. To address distressing internal world experiences, clinicians can enhance the person’s sense of safety, encourage healthy internal communication, or even employ hypnotic or imagery techniques.

## 6. Visions or Voices “Produced” by Dissociated Self-states

In some cases, the alter may communicate with the “host” personality in some unexpected ways. They may, for example, produce some images to threaten the host. This could result in psychotic episodes [12], although it may later be discovered that the hallucinations or delusions are “produced” by an alter, who may have some unaddressed needs. Although these episodes may involve a loss of reality testing, resulting in psychotic episodes, they are fundamentally driven by dissociative processes.

## 7. The Delusion of Being a Separate Person (e.g., Dissociative Possession, Being an External/outside Agent) 

In the case of dissociative identity disorder or its partial form, some alters may perceive themselves as distinct individuals separate from the person. In some cases, this may manifest as experiences resembling ghost- or spirit-possession. In other cases, the alters may believe they are living in the 1990s, despite the current time being the 2020s, or they may perceive themselves as young children, even though the body is now that of an adult. These experiences are not true psychotic delusions and should be addressed using a trauma- and dissociation-informed approach.

## 8. Some Alter Personality States That are Psychotic

In dissociative identity disorder, it is possible that different alters may have different presenting symptoms, often with different underlying psychological meanings. In some cases, one alter is psychotic, whereas other alters are not. In other cases, all alters are psychotic. It is also possible that the symptoms of one alter may influence other alters. For example, a very depressed child alter can make the host feel depressed too. The same may be true for psychotic experiences. It is important to assess whether only some or all alters are psychotic, and if any of the alters inside may actually know the reasons or meanings behind the symptoms. It is true that, in some cases, dissociative individuals may have genuine psychotic episodes (e.g., dissociative psychosis), especially under extreme stressors, triggers, or severe internal conflicts [12, 13]. Usually, in the cases of complex dissociative disorders, dissociative psychosis episodes are stress-related and short-term. However, if the underlying causes continue to exist (e.g., the stressor is ongoing, the person and their alters do not feel safe, or the internal conflicts cannot be resolved), the mental health status of the person might unfortunately turn into long-term psychosis, and dissociation remains unrecognized behind the presenting psychotic symptoms.

By looking at these clinical features, trauma-related dissociative processes or symptoms may appear closely linked to psychosis and its symptoms. We suggest that trauma and dissociation be regularly and comprehensively assessed in people who present with psychotic symptoms because the differential diagnosis could have significant implications for treatments. If the individual suffers from trauma and dissociation but only their psychotic symptoms are recognized and treated, the root causes of the symptoms remain unaddressed, and the treatment may be ineffective. This may even result in unnecessary polypharmacy and side effects.
